# Delayed Diagnosis of Congenital Myasthenic Syndromes Erroneously Interpreted as Mitochondrial Myopathies

**DOI:** 10.3390/jcm12093308

**Published:** 2023-05-06

**Authors:** Mariana I. Muñoz-García, María Paz Guerrero-Molina, Carlos Pablo de Fuenmayor-Fernández de la Hoz, Laura Bermejo-Guerrero, Ana Arteche-López, Aurelio Hernández-Laín, Miguel A. Martín, Cristina Domínguez-González

**Affiliations:** 1 Neuromuscular Unit, Neurology Department, Hospital Universitario 12 de Octubre, 28041 Madrid, Spain; marianaisabel.munoz@salud.madrid.org (M.I.M.-G.); carlosdefuenmayor@hotmail.com (C.P.d.F.-F.d.l.H.);; 2 Genetics Department, Hospital Universitario 12 de Octubre, 28041 Madrid, Spain; 3 Neuropathology Department, Hospital Universitario 12 de Octubre, 28041 Madrid, Spain; 4 Spanish Network for Biomedical Research in Rare Diseases (CIBERER), 28041 Madrid, Spain; 5 Mitochondrial and Neuromuscular Diseases Research Group, 12 de Octubre, Hospital Research Institute (imas12), 28041 Madrid, Spain

**Keywords:** congenital myasthenic syndromes, mitochondrial myopathies, genetics, neuromuscular disorders

## Abstract

Background: Congenital myasthenic syndromes (CMSs) and primary mitochondrial myopathies (PMMs) can present with ptosis, external ophthalmoplegia, and limb weakness. Methods: Our method involved the description of three cases of CMS that were initially characterized as probable PMM. Results: All patients were male and presented with ptosis and/or external ophthalmoplegia at birth, with proximal muscle weakness and fatigue on physical exertion. After normal repetitive nerve stimulation (RNS) studies performed on facial muscles, a muscle biopsy (at a median age of 9) was performed to rule out congenital myopathies. In all three cases, the biopsy findings (COX-negative fibers or respiratory chain defects) pointed to PMM. They were referred to our neuromuscular unit in adulthood to establish a genetic diagnosis. However, at this time, fatigability was evident in the physical exams and RNS in the spinal accessory nerve showed a decremental response in all cases. Targeted genetic studies revealed pathogenic variants in the *MUSK*, *DOK7*, and *RAPSN* genes. The median diagnostic delay was 29 years. Treatment resulted in functional improvement in all cases. Conclusions: Early identification of CMS is essential as medical treatment can provide clear benefits. Its diagnosis can be challenging due to phenotypic overlap with other debilitating disorders. Thus, a high index of suspicion is necessary to guide the diagnostic strategy.

## 1. Introduction

Congenital myasthenic syndromes (CMSs) are a group of rare and heterogeneous neuromuscular junction disorders. Epidemiological data on CMS are scarce, but estimations set the total prevalence to around 3.2 cases per million of the total population in Spain [1] and 9.2 cases per million of under 18 years of age in the UK [2], with a male predominance (2:1) [3]. However, these are most likely underestimations because of mild phenotypes that are missed or confused with one of the many differential diagnoses. A review estimated the prevalence of CMS as 1/10 of total myasthenia gravis cases, which would result in a total prevalence of 25–125 cases per million [4].

Initially, the CMSs were classified according to the location of the altered protein as presynaptic, synaptic, basal-lamina-associated, or postsynaptic [5]. However, the expansion of gene sequencing has allowed the identification of over 30 genes associated with CMS, resulting in further subclassifications. As an example, presynaptic defects (representing 6% of total cases) can affect the synthesis and recycling of acetylcholine or the exocytosis of the synaptic vesicles [5,6]. The most prevalent presynaptic defect is the choline acetyltransferase deficiency (ChAT syndrome), which accounts for nearly 5% of total cases. Synaptic defects (compromising 15% of total cases) include endplate acetylcholinesterase deficiency caused by *COLQ* mutations (13%) and the basal lamina defects, which are less frequent [5,6,7].

Postsynaptic syndromes represent around 50% of all defects. These syndromes include mutations of the acetylcholine receptor (AChR), which can affect the number of receptors, and are most commonly associated with mutations in the E subunit (*CHRNE* mutations). Other mutations impact the kinetic function of the receptor, resulting in either fast channel CMS (where receptor opening times are reduced) or slow channel CMS (where receptor opening times are increased). Additionally, defective receptor clustering and synaptic stability are sometimes categorized as post-synaptic syndromes or grouped in a different category altogether [5,6,7].

This latter group constitutes approximately 25–35% of all cases and involves defects in the AGRN-LRP4-MUSK-DOK7 signaling pathway, which is crucial in the neuromuscular junction (NMJ) formation, structure, and maintenance [5,6,7]. Agrin, the product of *AGRN*, binds to a lipoprotein-related protein (LRP4) in the postsynaptic membrane. The agrin-LRP4 complex binds to and activates the muscle–skeletal receptor tyrosine kinase (encoded by *MUSK*).

Activated MUSK phosphorylates downstream of kinase 7 (encoded by *DOK7*), which leads to the recruitment of two adaptor proteins that serve as activators. Full activation of MUSK also results in activation of rapsyn (encoded by *RAPSN*), which concentrates acetylcholine receptor (AchR) on the postsynaptic membrane and promotes postsynaptic differentiation [5]. The *RAPSN* gene is the most mutated in this group, representing 15–20% of all cases, followed by *DOK7* (10–15%) [7]. Additionally, around 4% of CMSs result from defects in protein glycosylation since the NMJ is heavily glycosylated, and glycosylation of the AChR subunits is necessary for assembly and transport to the cellular membrane, resulting in pre-synaptic and post-synaptic transmission deficits [5,6]. Finally, other CMSs have been described in association with defects in nuclear membrane proteins, intermediate filaments (plectin), and sodium channel deficiencies, or associated with congenital myopathies, and even defects in the mitochondrial citrate carrier [5].

The general clinical picture is defined by fatigable and fluctuating muscular weakness due to a defect in neuromuscular transmission, but the phenotypes vary significantly. The age of onset is generally in the first decade of life. Still, it sometimes presents in the second or third decade, with a wide range of severity, progression (progressive, fluctuating, or regressive), and patterns of muscular weakness, typically involving ocular, bulbar, and limb muscles [8]. There are certain diagnostic clues that can help identify specific syndromes. For instance, sudden apneic episodes triggered by fever or stress may suggest *ChAT*, *RAPSN*, or sodium channel myasthenia, while the presence of nephrotic syndrome may indicate basal lamina defects [5].

The diverse phenotypes and presentations of CMS lead to a broad differential diagnosis, which differs between the childhood-onset CMS and the adult-onset CMS. In childhood-onset CMS, it is often challenging to distinguish between CMS and congenital myopathies, primary mitochondrial myopathies (PMMs), and autoimmune myasthenia gravis. In the adult-onset CMS, the differential diagnosis also encompasses certain forms of motor neuron disease or peripheral neuropathy, as well as PMM, in most cases [5].

PMMs are a heterogeneous group of genetic disorders caused by defects in oxidative phosphorylation, predominantly affecting skeletal muscle. Patients with PMM may experience fluctuating symptoms, such as exercise intolerance and fatigue, as well as ocular manifestations, such as ptosis and progressive external ophthalmoplegia [8]. This overlap of symptoms between CMS and PMM can cause clinical confusion, underscoring the importance of complementary studies to achieve an accurate diagnosis. However, it is essential to interpret such ancillary tests in conjunction with clinical information to avoid misinterpretation. In this work, we aim to describe three cases of congenital myasthenic syndromes initially misdiagnosed as mitochondrial syndromes and only correctly classified as adults.

## 2. Materials and Methods

We describe the clinical and complementary data, obtained as standard evaluations within the healthcare diagnostic process, of three patients with CMS who were referred to the neuromuscular unit of the Hospital Universitario 12 de Octubre in Madrid, Spain, a national reference center, previously diagnosed as probable PMM. Patients gave their informed consent to publish their clinical data anonymously. The research conducted was approved by the Ethic Committee of our center (Comité de Ética de la Investigación con medicamentos del Hospital Universitario 12 de Octubre; approval code, 18/487; approval date, 27 November 2018).

## 3. Results

The information of the diagnostic studies performed before our evaluation and, subsequently, at our neuromuscular unit for each case is shown in Table 1.

### 3.1. Patient 1

A 50-year-old man presented to our clinic with a previous diagnosis of probable PMM, with chronic progressive external ophthalmoplegia (CPEO) phenotype. His family history for neuromuscular disorders, cardiopathies, or eye disorders was negative, and no consanguinity was reported. He had surgery at 38 years old to correct bilateral ptosis and presented an external ophthalmoplegia of unknown onset, without binocular diplopia (probably congenital). He also described a history of early fatigue and proximal and distal limb weakness after sustained physical activity that began around 30 years old, without episodes of rhabdomyolysis.

A neurological examination revealed surgically corrected ptosis, incomplete abduction, and adduction of eye movements bilaterally, as well as fixed symmetrical proximal and distal weakness in the upper limbs (Medical Research Council (MRC) grade 4/5 in elbow flexion, finger extension, and interossei muscles).

Complementary studies performed in other centers (see Table 1) revealed a slightly elevated creatinine kinase (400 UI/L, normal < 170) and a myopathic EMG with abnormal jitter in facial muscles and normal repetitive nerve stimulation (RNS). The specifics of the neurophysiological examination were not available. A muscle biopsy showed <1% cytochrome c oxidase (COX)-negative fibers without other histological morphological alterations (performed at age 48). No defects in respiratory chain activity were identified, and the most frequent point mutations and a large-scale single deletion in the mitochondrial DNA (mtDNA) extracted from the muscle were ruled out.

In our clinic, we conducted an examination to evaluate clinical fatigability, which showed an increase in weakness in the flexion of the neck and upper and lower limbs after repeated maneuvers. Subsequently, we performed a new neurophysiological study targeting weak muscles of the upper extremities. We conducted RNS at a low frequency (3 Hz) in the spinal accessory and ulnar nerves, and it revealed a decremental response of 20% in the trapezius and 15% in the abductor digiti minimi (ADM), respectively. Since the RNS study results were abnormal, we did not perform a jitter study.

An analysis of a phenotype-driven virtual gene panel directed toward CMS after exome sequencing (ES) revealed two predicted missense variants in the *MUSK* gene: a c.114T>A (p.Asp38Glu), which had already been described as pathogenic [9]; and a c.251T>C (p.Leu84Pro), which was classified as a variant of uncertain significance according to the American College of Medical Genetics and Genomics (ACMG) criteria [10]. A segregation study among his three asymptomatic siblings was performed. Two were heterozygous carriers of the c.114T>A variant, whereas none harbored the second variant. A brief description of this case was previously published to describe this variant [11].

The patient was initially treated with 50 mg of ephedrine and 40 mg of 3,4-diaminopyridine (3,4-DAP) per day, with clear clinical improvement: weakness and fatigability were no longer evident in the axial or proximal muscles. However, no improvement was noted in the ophthalmoplegia. During follow-up, the patient discontinued the 3,4-DAP without worsening of symptoms, and a short switch to salbutamol did not correlate with an equivalent benefit as with ephedrine. After 8 years of treatment with 50 mg of ephedrine per day, the patient remains stable, maintaining the benefit of treatment and without any adverse effects.

### 3.2. Patient 2

A 29-year-old male was referred to our clinic with a PMM diagnosis. His family history was unrevealing. He was born at term to non-consanguineous parents, and there were no perinatal complications. His developmental milestones were age-appropriate, but bilateral non-fluctuating ptosis and early fatigue, along with frequent falls, were noted from age 3 to 4. He had corrective palpebral surgery at 20 years old, and this resulted in defective ocular closure. Previous neurological examinations described the presence of mild facial and proximal lower-limb weakness, in addition to ptosis.

A muscle biopsy was performed at age 4, reporting the presence of some abnormal mitochondria. The respiratory chain analysis revealed a slight single defect of complex I activity estimated as NADH dehydrogenase at that time (78% of the lower limit of controls) [12]. The workup also included EMG, cardiological exams, and spirometry studies, which were all normal. Treatment with vitamins and antioxidants provided no benefit.

The initial history at our clinic did not suggest any symptoms of fatigability, dysphagia, visual alterations, or other systemic symptoms. The neurological exam during this first visit revealed partial ophthalmoparesis and bilateral facial weakness but no limb or axial weakness or signs of fatigability. The muscle magnetic resonance image (MRI) was normal. Genetic studies targeting frequent pathogenic variants in mtDNA showed no abnormalities.

However, during a follow-up visit, the neurological exam revealed limb girdle symmetrical weakness (MRC grade 4/5), which prompted a new neurophysiological study focusing on the weakened muscles in the upper extremities. Low-frequency (3 Hz) RNS was carried out on the spinal accessory nerve, revealing a 15% decremental response in the trapezius muscle. However, the RNS response in ADM after stimulating the ulnar nerve was normal. Given the abnormal RNS results, a jitter study was not performed.

An analysis of a CMS ES-virtual panel revealed two previously described frameshift pathogenic variants in the DOK7 gene: c.1120_1121insGCCT (p.Ala378SerfsTer29) and c.1373dupC (p.Gln460ProfsTer58) [13]. His father resulted in being heterozygous only for the c.1124_1127dup (p.A378Sfs*29) variant, supporting the presence of the variants in compound heterozygosity in the patient.

The patient experienced significant clinical improvement after the initiation of treatment with salbutamol. He eventually needed only between 2 and 4 mg per day. The surgical correction of the ptosis was reversed at the patient’s request, without the appearance of relevant ptosis.

### 3.3. Patient 3

A 29-year-old male with a clinical diagnosis of PMM requested a second opinion at our clinic. His family history was negative, and no consanguinity was reported. He was born with severe hypotonia, and during his first years of life, he presented several episodes of apnea and respiratory failure that required intensive care. Previous medical reports described ophthalmoparesis and fatigable bilateral ptosis but no diplopia.

During follow-ups in other medical centers, an extensive workup was conducted (see Table 1). An initial muscle biopsy performed at 3 years of age showed no relevant morphological changes, but an analysis of the respiratory chain activity performed on muscle tissue reported complex IV deficiency. At 12 years of age, a second biopsy was performed, and in this case, the histology and the analysis of the respiratory chain activity were reported as normal.

On the first visit to our clinic, the patient reported intense muscular fatigue, with noticeable fluctuations from day to day and worsening episodes triggered by stress or prolonged physical activity. He did not report muscle pain or episodes suggestive of myoglobinuria, dysphagia, or other neurological or systemic symptoms.

The neurological exam showed bilateral asymmetrical ptosis with a positive Cogan sign, ophthalmoparesis with restriction to vertical movements and adduction bilaterally, and fatigable cervical and upper-limb symmetrical weakness (MRC grade 4/5 before and 3/5 after fatigability maneuvers in proximal muscles in upper limbs). He could not jump or run or get up from the floor or a chair without support. A neurophysiological study was conducted to examine the weak upper limb muscles, which revealed a pathological low-frequency (3 Hz) RNS with a decrement of 17% in the trapezius muscle after spinal accessory nerve stimulation. Additionally, a 13% decremental response was observed in ADM after stimulating the ulnar nerve.

A genetic analysis of a CMS ES-virtual gene panel identified two pathogenic variants in the *RAPSN* gene: a frameshift c.1185del, p.(Thr396ProfsTer12) variant, which was of maternal origin; and a predicted missense c.264C>A, p.(Asn88Lys) variant, which was of paternal lineage. The first variant was previously reported as pathogenic and was proposed as a founder mutation in the Spanish population [14]. The second variant was associated with CMS in both homozygosis and compound heterozygosis [15].

The patient maintains treatment with 360 mg of pyridostigmine daily, with clear, incomplete clinical benefit. Worsening episodes triggered by stress or infections persist, and adding 60 mg of 3,4-DAP does not improve the condition significantly.

## 4. Discussion

We report three cases of CMS that were initially misdiagnosed as primary mitochondrial myopathies. Both entities can present with ptosis, ophthalmoplegia, and muscle weakness of variable onset. Furthermore, patients with CMS may have mild elevated CK levels and myopathic changes in EMG studies, which may falsely lead to a diagnosis of myopathy [3]. However, fluctuations in muscle weakness and, especially, fatigability upon examination are the most relevant clinical characteristics that should direct the differential diagnosis toward neuromuscular junction transmission defects. The muscle-biopsy findings and the results of other complementary tests should always be interpreted in conjunction with the clinical context. The importance of identifying CMS relies on the potential benefit of symptomatic treatment.

General descriptions of each defect’s clinical phenotype vary significantly, demonstrating the extensive variability of CMS. However, certain previously described characteristics of each type of defect are seen in our patients.

CMSs due to *MUSK* mutations have a broad phenotype, including fetal akinesia, isolated vocal cord paralysis, and late-onset limb girdle weakness with variable ocular involvement. A common feature in this group of patients is the ineffectiveness or worsening after treatment with acetylcholinesterase (AChE) inhibitors, and this is why treatment with ephedrine and 3,4-DAP was preferred in our patient [7]. 3,4-DAP is a potassium channel blocker that increases acetylcholine release. Ephedrine is an adrenergic agonist and improves symptoms through a poorly understood mechanism [5,16].

*DOK7* mutations are associated with a predominantly limb–girdle pattern of weakness that is accompanied by nonspecific myopathic features. The onset of symptoms is characterized by a waddling gait due to muscle weakness in childhood, after achieving normal development motor milestones, as was also noted in our patient. In early onset cases, the presence of stridor and the observation of tongue wasting may serve as specific clues [17]. Ptosis is frequent but not external ophthalmoplegia. Fatigability can be absent, but prolonged periods of weakness may occur [4,7]. In this group, AChE inhibitors can also worsen the symptoms, but adrenergic beta-agonists are usually effective, with a slow and steady response over time [5,16,17]. In fact, the response to B2-agonist medication was first described following the identification of *DOK7* mutations as a major cause of CMS [16].

*RAPSN* mutations are responsible for a CMS where an early onset presentation may include malformations present at birth (arthrogryposis), respiratory distress, hypotonia, and poor feeding. However, late-onset milder manifestations with generalized or limb weakness and ptosis may also be observed, but ophthalmoplegia is rare. In a review of 10 patients with *RAPSN* mutations, it was suggested that diplopia without clear ophthalmoparesis could serve as a reliable criterion for differentiating rapsyn-CMS mutations from CMS caused by mutations affecting the number of AChR expressed in the NMJ [14]. Respiratory failure and fluctuations in the setting of infections and stress are reported frequently in this phenotype, especially at an early age and becomes less common as the patient gets older [4,6,7]. However, respiratory crises are not specific to *RAPSN* defects and can be seen in any CMS [3]. On the contrary, fluctuations in weakness of the cervical and proximal limb girdle muscles, along with bulbar symptoms and ptosis, are believed to be more specific symptoms of this subtype of CMS, and these symptoms can persist throughout the patient’s lives [14]. The response to AChE inhibitors is favorable and can be improved by adding 3,4-DAP. Treatment can improve both the muscular symptoms and the frequency of exacerbations. Both AChE inhibitors and 3,4-DAP increase the endplate potential amplitude to meet the neuromuscular transmission safety margin by increasing the amount of acetylcholine at the NMJ [5]. The recommended dosage for these patients is around 200 mg/day in adults or 5–10 mg/kg/day in children, and adding 3,4-DAP typically results in a 30% reduction in pyridostigmine dosage [14]. However, our patient required higher doses, and the addition of 3,4-DAP did not show any significant benefits.

All forms of CMS that result from the AGRN-LRP4-MUSK-DOK7 signaling pathway for clustering of the AchR appear to have a positive response to B2-agonists. The response, however, is not immediate, unlike myasthenia gravis or CMS that respond to AChE inhibitors [16].

Research suggests that B2 agonists can improve the structure of the neuromuscular junction [18]. In studies involving *DOK7* knockout mice models, treatment with B2-agonists resulted in an increase in the number of active NMJs, suggesting enhanced stability [18]. Although little is known about the molecular mechanism behind this effect, it has been observed that adrenergic receptors are present in higher densities at the postsynaptic membrane, and there is evidence that the NMJ may receive direct sympathetic innervation [19]. Therefore, it is likely that the B2-adrenergic receptor plays a role in maintaining or enhancing the postsynaptic structure of the NMJ junction.

Interestingly, there are currently no reported cases of patients with *RAPSN* mutations experiencing improvement with B2-agonists. However, given the evidence suggesting that these drugs can stabilize the NMJ, it is possible that they could also be beneficial for these patients. Other treatments used in CMS include open-channel blockers, such as fluoxetine and quinidine, which are typically used to treat slow-channel mutations and have a lesser effect on other mutations [16]. Recently, two cases of steroid-responsive *DOK7* CMS were reported [20]. Future treatment strategies may include gene therapy using adenovirus-associated vectors [21], antibodies that activate MUSK, or small molecules that aim to stabilize the NMJ [6,22].

Neurophysiological studies can direct the differential diagnosis and differentiate a muscle disorder from an NMJ disorder. The sensitivity of RNS in CMS has been described in the range of 65–90.5%, with a high specificity that depends on the muscle tested. For example, a study found a 98.1% specificity with a protocol that includes six muscles: two facial muscles, two proximal muscles, and two distal muscles [23]. However, the best logical approach would be to directly test a weak muscle and, if necessary, repeat studies.

The sensitivity of jitter measurement is even higher, ranging from 85% to 95.2%, with a specificity of 86.5% or higher depending on the cutoff for mean jitter values. However, jitter abnormalities can also be found in CPEO or congenital myopathies. It seems that the combination of both abnormal RNS and abnormal jitter tests has the greatest accuracy for distinguishing CMS from CPEO and congenital myopathies [23]. In our experience, the accuracy of this test can be affected by the examiner’s level of expertise, which can lead to confusion in some cases. Therefore, it is crucial to consider the entire clinical picture when interpreting the results of the neurological exam and RNS. In some cases, a pathological neurological exam and RNS may be sufficient to guide genetic studies. In addition, it’s important to note that other findings, such as an EMG reported as myopathic, are not specific and should be interpreted within the appropriate context.

No specific findings on muscle biopsies are known for any of the CMSs. Most patients have normal biopsies, although some myopathic signs can be found, such as fiber-size variability, mild dystrophic features, or lipidosis [4]. Primary mitochondrial oxidative phosphorylation defects often show scarce ragged red fibers and/or COX-deficient fibers on muscle biopsy. However, these findings are neither mandatory nor specific to primary mitochondrial diseases. Both features can also be found in normal aging and other neuromuscular disorders [8].

The findings of respiratory chain defects in two of the patients could be related to technical errors [24] or secondary defects due to an alteration in NMJ proteins, as has been suggested in other similar reports [25]. In any case, the presence of COX-negative fibers or isolated MRC defects without genetic confirmation of pathogenic variants in nuclear or mtDNA should not lead to a diagnosis of mitochondrial disease. The genetic diagnosis of mitochondrial disorders is highly complex due to their heterogeneous nature and dual genetic control. Recently, sensitive, and specific biomarkers that aid in this diagnosis, such as GDF15 levels, were described. These biomarkers can help identify which patients would benefit from performing more advanced studies to confirm the diagnosis [26]. However, the aim of this article is not to detail the diagnostic strategy for suspected cases of mitochondrial disease, we just want to emphasize that a diagnosis of “possible PMM” should not be made without sufficient evidence. If the underlying genetic defect is not known, this diagnosis should always be questioned. Otherwise, there is a risk of significantly delaying the correct diagnosis.

Although we focused on the differential diagnosis between CMS and mitochondrial myopathies, other entities, such as seronegative myasthenia gravis, congenital myopathies, or muscular dystrophies, must be considered. The importance of establishing a reliable diagnosis of CMS lies in the possibility of offering an effective treatment that depends on the subtype of CMS, which, in turn, will be typified by genetic confirmation.

## 5. Conclusions

CMS is likely an underdiagnosed condition due to its broad clinical spectrum, which includes PMM, in both children and adults, presenting with variable ptosis, ophthalmoplegia, and fluctuating muscular weakness. Conducting a directed neurological examination to look for signs of fatigability, along with a neurophysiological study with RNS directed at weak muscles, is crucial to guide genetic studies toward CMS.

Diagnosing CMS is essential because symptomatic and stabilizing treatments are available which can significantly improve the quality of life of affected patients.

## Figures and Tables

**Table 1:** Primary and secondary diagnostic workup in the cases of congenital myasthenic syndrome initially diagnosed as probable primary mitochondrial myopathies.

	Patient 1	Patient 2	Patient 3
Primary workup (performed at other medical centers)			
Sex	Male	Male	Male
Age of first symptoms	At birth	3–4	At birth/infancy
Signs and symptoms	Congenital external ophthalmoplegia and bilateral ptosis. Limb–girdle weakness at 30yo. Exercise intolerance.	Fixed palpebral ptosis, motor clumsiness and exercise intolerance	Generalized hypotonia, ptosis, episodes of respiratory insufficiency.
Blood analysis	CK: 400 U/L (34–171 U/L)	CK, aldolase, pyruvate, lactate: normal (values not available)	CK: 158 U/L (34–171 U/L)
EMG-ENG	Myopathic, abnormal jitter in facial muscles with normal RNS	Normal RNS in facial nerve.	Myopathic. Normal RNS
Muscle biopsy (age)	Fibers with size variability. A total of 3 cytochrome c oxidase-negative fibers (<1% of total). No MRC defects. (48)	Fibers size variability, type I fiber predominance, isolated megamitochondria. No ragged red fibers. Complex I defect. (5)	1: MRC complex IV defect (3) 2: MRC complex I in the lower limit (12)
Secondary workup (performed at H12O specialized neuromuscular unit)			
Age	50	29	29
Signs and symptoms	Surgically corrected ptosis. Ophthalmoplegia. Axial weakness. Proximal fatigability	Surgically corrected ptosis, partial ophthalmoparesis, bilateral symmetrical facial weakness. Fatigability and proximal limb–girdle weakness	Fatigable asymmetrical ptosis, incomplete ophthalmoparesis, strabismus, and fatigable axial and proximal limb muscle weakness.
EMG-ENG	Pathological RNS in spinal accessory and ulnar nerves	Pathological RNS in spinal accessory, normal in ulnar nerve	Pathological RNS in spinal accessory
Altered gene	*MUSK* (NM_005592.3)	*DOK7* (NM_173660.4)	*RAPSN* (NM_005055.4)
Variant 1	c.114T>A [p.(Asp38Glu)] → Pathogenic	c.1120_1121insGCCT [p.(Ala378SerfsTer29)] → Pathogenic	c.1185del [p.(Thr396ProfsTer12)] → Pathogenic
Variant 2	c.251T>C [p.(Leu84Pro)] → VUS	c.1373dupC [p.(Gln460ProfsTer58)] → Pathogenic	c.264C>A [p.(Asn88Lys)] → Pathogenic
Treatment	Ephedrine and 3,4-diamnipyridine	Salbutamol	Pyridostigmine and 3,4-diamnipyridine
Response	Improvement	Improvement	Improvement

CK, creatine kinase; EMG-ENG, electromyography–electroneurography; RNS, repetitive nerve stimulation; MRC, mitochondrial respiratory chain; H12O, Hospital 12 de Octubre; VUS, variant of unknown significance.

## Data Availability

The data in this study are available on request from the corresponding author. The data are not publicly available due to privacy restrictions.
